# The effect of comorbidity on the use of adjuvant chemotherapy and survival from colon cancer: a retrospective cohort study

**DOI:** 10.1186/1471-2407-9-116

**Published:** 2009-04-20

**Authors:** Diana Sarfati, Sarah Hill, Tony Blakely, Bridget Robson, Gordon Purdie, Elizabeth Dennett, Donna Cormack, Kevin Dew

**Affiliations:** 1 Department of Public Health, University of Otago Wellington, PO Box 7343, Wellington 6242, New Zealand; 2 Department of Society, Human Development and Health, Harvard School of Public Health, Harvard University, Boston, MA 02115, USA; 3 Department of Surgery, University of Otago Wellington, PO Box 7343, Wellington 6242, New Zealand; 4 Department of Sociology, Victoria University, PO Box 600, Wellington 6140, New Zealand

## Abstract

**Background:**

Comorbidity has a well documented detrimental effect on cancer survival. However it is difficult to disentangle the direct effects of comorbidity on survival from indirect effects via the influence of comorbidity on treatment choice. This study aimed to assess the impact of comorbidity on colon cancer patient survival, the effect of comorbidity on treatment choices for these patients, and the impact of this on survival among those with comorbidity.

**Methods:**

This retrospective cohort study reviewed 589 New Zealanders diagnosed with colon cancer in 1996–2003, followed until the end of 2005. Clinical and outcome data were obtained from clinical records and the national mortality database. Cox proportional hazards and logistic regression models were used to assess the impact of comorbidity on cancer specific and all-cause survival, the effect of comorbidity on chemotherapy recommendations for stage III patients, and the impact of this on survival among those with comorbidity.

**Results:**

After adjusting for age, sex, ethnicity, area deprivation, smoking, stage, grade and site of disease, higher Charlson comorbidity score was associated with poorer all-cause survival (HR = 2.63 95%CI:1.82–3.81 for Charlson score ≥ 3 compared with 0). Comorbidity count and several individual conditions were significantly related to poorer all-cause survival. A similar, but less marked effect was seen for cancer specific survival. Among patients with stage III colon cancer, those with a Charlson score ≥ 3 compared with 0 were less likely to be offered chemotherapy (19% compared with 84%) despite such therapy being associated with around a 60% reduction in excess mortality for both all-cause and cancer specific survival in these patients.

**Conclusion:**

Comorbidity impacts on colon cancer survival thorough both physiological burden of disease and its impact on treatment choices. Some patients with comorbidity may forego chemotherapy unnecessarily, increasing avoidable cancer mortality.

## Background

Comorbidity is the co-existence of diseases or disorders in addition to a primary disease of interest. Comorbidity has a detrimental effect on cancer survival, and its effect varies by cancer site [1-10]. Colon cancer is a disease of older people, many of whom have concomitant medical conditions at diagnosis. A number of studies have suggested that high levels of comorbidity are associated with poorer survival among colon cancer patients [1,3,4,11-15]. However, most of these studies have not disentangled the direct effect of comorbidity on survival, with the potential indirect impact of comorbidity on treatment choice which, in turn, may impact survival. These studies have also tended to have an exclusive focus on all-cause survival, reliance on administrative data and/or have used only one method to measure comorbidity.

Understanding the role of comorbidity in cancer survival helps clinicians assess an individual's prognosis and provides researchers with tools to allow them to stratify or adjust groups of patients according to risk in the same way they do for demographic and disease factors such as age and tumour stage [2]. Measuring comorbidity is not straight forward and a variety of approaches have been used including counts of conditions, comorbidity indices, and analysis of specific conditions [2,6-8,15-20]. Simple counts are problematic because they take no account of the seriousness of different underlying conditions. Comorbidity indices attempt to address this in various ways by combining both the number and seriousness of concomitant conditions into a single numeric score. This approach still has limitations however, since different conditions and combinations of conditions impact outcomes differently depending on both the primary condition of interest (e.g. breast cancer versus lung cancer) and the outcome being measured (e.g. mortality versus inpatient costs). Using individual conditions is also problematic because of the potentially large number of variables that need to be included in the analysis and the lack of an overall global measure of comorbidity.

It is well established that treatment with adjuvant chemotherapy after resection of stage III colon cancer improves survival [21-24]. There is also good evidence that the benefits of adjuvant therapy do not diminish with increasing age [22,25-28]. Yet older people and those with comorbidity are less likely to receive optimum adjuvant treatment for cancer [7,11,25,27,29-32]. This may be because clinicians are concerned that advanced age and concomitant conditions may increase the toxicity and side effects of treatment, that treatments may be less effective in these groups, or that the life expectancy of these patients is insufficient to justify the use of potentially toxic agents [11,25,27,33,34]. It is also possible that these patients themselves are more likely to decline treatment [33-35].

This study investigates the impact of comorbidity on both cancer specific and all-cause survival among colon cancer patients using detailed comorbidity data extracted from clinical notes. We assessed three different measures of comorbidity – comorbidity count, the Charlson Index [18] and individual conditions – in terms of how well they predict cancer specific and overall survival. Finally, we assessed the extent to which comorbidity-related differences in survival are mediated by different treatment options by assessing how comorbidity affects the offer of adjuvant chemotherapy among those with stage III colon cancer, and the extent to which the offer of chemotherapy affects cancer specific and overall survival.

## Methods

New Zealand residents diagnosed with colon cancer between 1996 and 2003 were eligible for study inclusion. Cases came from patients notified to the New Zealand Cancer Registry with a primary tumour in the colon (ICD-10-AM site codes C18-C19 excluding 18.1) and morphology consistent with adenocarcinoma. (New Zealand has mandatory registration of all primary cancers except non-melanoma skin cancers and carcinoma-in-situ.) Patients were ineligible if they were less than 25 years at diagnosis, were normally resident outside New Zealand, had a previous diagnosis of colon cancer, or were diagnosed after death. All Māori patients meeting the above criteria were included along with an approximately equal number of randomly-sampled non-Māori patients. This random sample was identified by assigning a unique random six-digit number to all non-Māori cases, placing these in numerical order and selecting the first four hundred (the approximate size of the Māori cohort). This sampling frame allowed an assessment of survival disparities between Māori and non-Māori patients with colon cancer (data presented elsewhere)[36].

Clinical data were abstracted from patients' clinical records from both public and private health care providers. Data were collected on all major comorbid conditions present at the time of diagnosis and all conditions included in the Charlson comorbidity index. The Charlson index was developed in 1987 using data from a cohort of 607 medical patients, and validated with a population of breast cancer patients. Nineteen conditions are allocated a weight of 1 to 6 depending on the adjusted relative risk of 1-year mortality, and summed to give an overall score [18]. The higher the individual's score, the higher the level of comorbidity. Additional conditions included in the comorbidity count were angina, hypertension, cardiac arrhythmias, previous pulmonary embolism, cardiac valve disease, inflammatory bowel disease, other neurological conditions (including multiple sclerosis, Parkinson's disease, epilepsy) and major psychiatric conditions (including schizophrenia, bipolar disease and depressive psychosis).

Pathology reports were obtained for all patients from their clinical records, the Cancer Registry or directly from the reporting laboratory. Data were recorded on a standardised form by a physician (SH) and double-entered into an electronic database. Data included patients' details (age, sex, ethnicity and small area deprivation), tumour characteristics (including location, histological features and stage at diagnosis), smoking status and treatment factors (such as receipt of surgery and chemotherapy offered). Small area deprivation was defined by the NZ Deprivation Index, an ecological measure based on a combination of nine socioeconomic variables derived from the national census (i.e. housing tenure, benefit receipt, unemployment, income, telephone access, car access, single parent families, education and household crowding). This was assigned according to each patient's domicile (census area) code at the time of diagnosis [37]. Stage was based on TNM staging groups [38].

Outcome data (vital status and cause of death) were obtained by linking study patients to the New Zealand national mortality database, with follow-up to the end of 2005. Patients whose deaths were not recorded in the mortality database were assumed to be still alive at the end of follow-up. For cancer specific analyses, patients who died from causes other than colon cancer were censored at the date of death.

Comorbidities were classed in three different ways: i) The total number of comorbid conditions ('comorbidity count') was summed for each patient and categorised into four groups 0, 1, 2 or 3+ conditions; ii) Charlson comorbidity scores were categorised into 0, 1–2 or 3+; and iii) Specific comorbid conditions were individually categorised.

Cox proportional hazards modelling was used to calculate hazard ratios (HRs) for cancer specific and all-cause mortality. Crude and adjusted HRs were compared for each of the three approaches to classifying comorbidity (i.e. comorbidity count, Charlson comorbidity index and specific conditions) to see how well each approach predicted survival. Hazard ratios were first adjusted for patient factors (age, sex, ethnicity, year of cancer registration, deprivation, smoking) and then adjusted for patient factors and disease factors (stage, grade and site of cancer).

To assess how well the three different measures of comorbidity predicted cancer specific and all-cause survival, the fit of a baseline model including patient and disease factors was compared to models that included each of the three approaches in turn using the likelihood ratio test. Individual conditions were included in the models in two ways; firstly by including only conditions with predictive HR of 1.2 or more, and secondly by including all individual conditions.

Adjuvant chemotherapy was recommended for use in patients with stage III colon cancer throughout the period of the study [39]. Logistic regression restricted to patients with stage III cancer was used to assess the relative odds of those with and without comorbidity being offered such therapy. Cox regression modelling was used to assess the impact on survival of the offer of chemotherapy after adjusting for age, sex, ethnicity and comorbidity.

All analyses were carried out in STATA (version 10) [40]. Approval for this study was granted by the New Zealand Multi-Region Ethics Committee.

## Results

A total of 776 patients met the study criteria based on Cancer Registry records. Ninety one (12%) of those sampled were later excluded because further information showed they were ineligible for study inclusion (65 had miscoded data (primarily cancer site) in the Cancer Registry and a further 26 had no histological diagnosis), giving 685 patients in total. Full data were obtained for 589 (86.0% of the eligible sample). During the follow up period 227 patients were identified as dying from colon cancer and 89 from other causes.

As expected, most colon cancer patients were aged over 55 years and were more likely to live in more deprived areas in New Zealand, consistent with the age structure of this cohort. Just under half the cohort was Maori, in keeping with the original sampling strategy. A fifth were current smokers, and a further 39% were ex-smokers. The disease related factors (stage, grade and site) were reasonably consistent with the largely unscreened population in New Zealand (Table 1). All patients received standard surgical treatment (tumour resection) except a small proportion (6%) who died before surgery or had clinically advanced disease for which surgery was not indicated.

**Table 1:** Characteristics of cohort participants

Patient factors		n	%
Sex	Male	305	51.8
	Female	284	48.2

Age	25–54 yrs	98	16.6
	55–64 yrs	154	26.2
	65–74 yrs	186	31.6
	75+ yrs	151	25.6

Ethnicity*	Maori	285	51.6
	Non-Maori	304	48.4

NZDep	1–2	59	10.0
	3–4	79	13.4
	5–6	111	18.9
	7–8	159	27.0
	9–10	181	30.7

Smoking	Non-smoker	234	39.7
	Ex-smoker	232	39.4
	Smoker	123	20.9

**Disease factors**		n	%

Stage	I	76	12.9
	II	183	31.1
	III	190	32.3
	IV	140	23.8

Grade	Well-differentiated	57	9.7
	Moderately	424	72.0
	Poorly	108	18.3

Site	Right	239	40.6
	Left	220	37.4
	Rectosigmoid	99	16.8
	Synchronous	31	5.3

**Comorbidities**		n	%

	Angina	72	12.2
	Hypertension	224	38.0
	Previous MI	49	8.3
	Cardiac arrhythmias	79	13.4
	CHF	62	10.5
	PVD	24	4.1
	Chronic respiratory disease	129	21.9
	GI ulcer disease	24	4.1
	Other cancer	27	4.6
	Cerebrovasc disease	41	7.0
	Diabetes (all)	95	16.1
	Renal disease (all)	31	5.3
	Other neurological	39	6.6

Charlson comorbidity index score	O	284	48.2
	1–2	240	40.8
	3+	65	11.0

Number of conditions**	0	181	30.7
	1	147	25.0
	2	117	19.9
	3+	144	24.5

*Maori purposively oversampled

**Included all conditions included in Charlson Comorbidity Index as well as angina, hypertension, cardiac arrhythmias, previous pulmonary embolism, cardiac valve disease, inflammatory bowel disease, other neurological conditions (including multiple sclerosis, Parkinson's disease, epilepsy) and major psychiatric conditions (including schizophrenia, bipolar disease and depressive psychosis)

Seventy percent of patients had at least one comorbid condition and a quarter had three or more (Table 1). The most common conditions were hypertension (38.0%), chronic respiratory disease (21.9%), and diabetes (16.1%). Nearly half (48.2%) of the patients had a Charlson score of zero, 40.8% scored 1–2 and the remaining 11.0% scored 3 or more.

Table 2 shows hazard ratios firstly for cancer specific then for all-cause mortality for the three different measures of comorbidity. In the fully adjusted models, hazard ratios increased with increasing levels of both the comorbidity count and the Charlson comorbidity index. For individual comorbid conditions, congestive heart failure and diagnosis of a non-cerebrovascular neurological condition were significantly related to worse colon cancer specific survival after adjustment for patient and disease factors (HR = 1.83, 95% CI 1.14–2.93 and 1.96 95% CI 1.12–3.42 respectively). A history of previous myocardial infarction, chronic respiratory disease, cerebrovascular disease, diabetes, and chronic renal disease also appeared to predict worse survival (HR>1.2), but did not reach the level of statistical significance. In contrast, gastrointestinal ulcer disease, a history of other previous cancer, and angina appeared to have a non-significant protective effect although confidence intervals were wide (HR = 0.76, 95% CI 0.31–1.87, 0.71, 95% CI 0.32–1.55 and 0.81, 95% CI 0.51–1.30 respectively).

**Table 2:** Hazard ratios for cancer specific and all cause mortality for patients with specified co-morbidities

	Crude HR		Adjusted for pt factors^1^		Adjusted for pt and disease factors^2^	
**Cancer specific mortality**						

Angina	0.74	0.48–1.15	0.75	0.48–1.19	0.81	0.51–1.30
Hypertension	0.97	0.74–1.27	0.96	0.72–1.28	0.92	0.69–1.23
Previous MI	1.23	0.77–1.97	1.30	0.81–2.11	1.49	0.90–2.46
Cardiac arrhythmias	0.82	0.54–1.25	0.88	0.57–1.36	1.17	0.74–1.83
CHF	1.13	0.74–1.75	1.10	0.70–1.72	1.83	1.14–2.93
PVD	0.64	0.26–1.55	0.71	0.39–1.75	1.18	0.48–2.93
Chronic respiratory disease	1.16	0.85–1.58	1.06	0.77–1.46	1.24	0.89–1.74
GI ulcer disease	0.49	0.20–1.18	0.50	0.21–1.22	0.76	0.31–1.87
Other cancer	0.77	0.36–1.63	0.79	0.37–1.71	0.71	0.32–1.55
Cerebrovasc disease	1.23	0.75–2.02	1.30	0.78–2.16	1.25	0.74–2.10
Diabetes (all)	1.18	0.84–1.68	1.14	0.79–1.63	1.27	0.88–1.84
Renal disease (all)	1.10	0.60–2.01	1.01	0.54–1.87	1.42	0.76–2.66
Other neurological	1.19	0.71–1.97	1.35	0.80–2.25	1.96	1.12–3.42

Charlson comorbidity index						
O	1.00		1.00		1.00	
1–2	1.26	0.96–1.66	1.22	0.91–1.64	1.32	0.98–1.77
3+	0.86	0.52–1.42	0.88	0.53–1.47	1.48	0.88–2.50

Number of conditions*						
0	1.00		1.00		1.00	
1	1.30	0.93–1.80	1.29	0.92–1.83	1.06	0.76–1.55
2	1.15	0.79–1.68	1.13	0.75–1.70	1.23	0.81–1.87
3+	1.00	0.69–1.45	1.01	0.67–1.53	1.33	0.87–2.04

**All-cause mortality**						

Angina	0.96	0.68–1.34	0.86	0.61–1.23	0.91	0.63–1.31
Hypertension	1.03	0.82–1.29	0.93	0.73–1.19	0.90	0.70–1.15
Previous MI	1.67	1.17–2.38	1.74	1.21–2.52	1.91	1.31–2.79
Cardiac arrhythmias	1.26	0.93–1.72	1.19	0.86–1.65	1.48	1.07–2.06
CHF	1.79	1.31–2.45	1.61	1.16–2.24	2.30	1.63–3.25
PVD	1.46	0.87–2.46	1.40	0.82–2.40	2.02	1.18–3.48
Chronic respiratory disease	1.29	0.99–1.66	1.12	0.85–1.46	1.26	0.96–1.66
GI ulcer disease	0.61	0.32–1.19	0.60	0.31–1.17	0.76	0.38–1.49
Other cancer	1.31	0.79–2.16	1.25	0.74–2.10	1.16	0.68–1.98
Cerebrovasc disease	1.36	0.91–2.04	1.28	0.85–1.93	1.27	0.84–1.94
Diabetes (all)	1.48	1.12–1.95	1.44	1.08–1.92	1.53	1.15–2.05
Renal disease (all)	1.52	0.96–2.39	1.32	0.83–2.10	1.79	1.12–2.86
Other neurological	1.44	0.97–2.16	1.58	1.05–2.38	2.02	1.32–3.11

CCI						
O	1.00		1.00		1.00	
1–2	1.68	1.32–2.14	1.55	1.20–2.00	1.60	1.24–2.07
3+	1.84	1.30–2.60	1.78	1.24–2.55	2.63	1.82–3.81

Number of conditions*						
0	1.00		1.00		1.00	
1	1.30	0.95–1.78	1.26	0.92–1.73	1.08	0.78–1.49
2	1.50	1.08–2.08	1.40	0.98–1.99	1.45	1.01–2.07
3+	1.73	1.28–2.35	1.63	1.17–2.28	2.00	1.41–2.82

^1 ^includes age, sex, ethnicity, NZdep, year of registration, smoking

^2 ^includes age, sex, ethnicity, NZdep, year of registration, smoking, stage, grade and site of cancer

* Included all conditions included in Charlson Comorbidity Index as well as angina, hypertension, cardiac arrhythmias, previous pulmonary embolism, cardiac valve disease, inflammatory bowel disease, other neurological conditions (including multiple sclerosis, Parkinson's disease, epilepsy) and major psychiatric conditions (including schizophrenia, bipolar disease and depressive psychosis)

Global measures of comorbidity were strongly associated with poorer overall survival. In the fully adjusted models Charlson comorbidity index scores of 1–2 and 3 or more were associated with hazard ratios of 1.60 (95% CI 1.24–2.07) and 2.63 (95% CI 1.82–3.81) respectively, compared with a Charlson score of 0. Likewise, a greater number of conditions were associated with poorer survival. Individual comorbid conditions showed a stronger association with all-cause mortality than cancer specific mortality. After adjusting for patient and disease factors, significantly poorer survival was seen with a history of previous myocardial infarction (HR = 1.91, 95% CI 1.31–2.79), cardiac arrhythmias (HR = 1.48, 95% CI 1.07–2.06), congestive heart failure (HR = 2.30, 95% CI 1.63–3.25), peripheral vascular disease (HR = 2.02, 95% CI 1.18–3.48), diabetes (HR = 1.53, 95% CI 1.15–2.05), chronic renal disease (HR = 1.79, 95% CI 1.12–2.86) and diagnosis of other significant neurological conditions (HR = 2.02, 95% CI 1.32–3.11). Chronic respiratory disease and cerebrovascular disease also appeared to be non-significantly negatively related, and GI ulcer disease positively related to survival.

Table 3 shows the likelihood ratio test results when each comorbidity measure was added to the survival model. For the cancer specific mortality risk, adding either the Charlson index or the comorbidity count to the baseline Cox regression model did not significantly improve model fit over and above demographic and disease factors (p = 0.12 and 0.57 respectively). Inclusion in the model of individual conditions with adjusted hazard ratios of >1.2 provided significantly better fit (LR chi2 (7) = 14.46; p = 0.04). Adjusting for comorbidity had a more marked effect on model fit for all-cause mortality hazard with inclusion of any of the three measures of comorbidity resulting in highly significant improvement in model fit compared with the baseline model.

**Table 3:** Results of likelihood ratio test; baseline model as comparison: cancer specific mortality and all-cause mortality

	Cancer specific mortality		All-cause mortality	
Model 1 (baseline + Charlson categories)	LR chi2(2) = 4.28	P = 0.12	LR chi2(2) = 27.56	P < 0.0001

Model 2 (baseline + comorbidity count)	LR chi2(3) = 2.03	P = 0.57	LR chi2(3) = 18.60	P = 0.0003

Model 3 (baseline + conditions with independent* HR≥1.2)^1^	LR chi2(7) = 14.46	P = 0.04	LR chi2(9) = 46.23	P < 0.0001

Model 4 (baseline + all conditions with 20 or more cases)	LR chi2(13) = 19.93	P = 0.10	LR chi2(13) = 52.66	P < 0.0001

Baseline model adjusts for following covariates: Age, sex, ethnicity, NZdep, smoking, year of registration, stage, grade and site.

*after adjustment for patient and colon cancer related factors

^1^For cancer specific mortality this included previous myocardial infarction, congestive heart failure, cerebrovascular disease, other neurological conditions, diabetes, renal disease, chronic respiratory disease. For all cause mortality this included all these conditions and also cardiac arrhythmias, and peripheral vascular disease.

To ascertain whether the survival disadvantage of patients with higher comorbidity was related to different treatment choices we investigated whether comorbidity was associated with different levels of chemotherapy use, and whether that in turn was related to poorer survival among those with comorbidity. The Charlson Index was used because we required a global measure of comorbidity for this analysis. There were 190 patients with stage III disease in our cohort, 129 (68%) of whom were offered chemotherapy. Table 4 shows the proportions of patients with stage III cancer who were offered adjuvant chemotherapy by sex, age, ethnicity and comorbidity. Older patients and those with higher comorbidity were considerably less likely to be offered chemotherapy. For example, 63% of those aged over 75 years were not offered adjuvant therapy compared with 20% of those aged 55–64 years. Likewise, 16% of those without any recorded comorbidity were not offered chemotherapy, compared with 81% of those with the highest level of comorbidity. The adjusted odds ratios (Table 4) show that age and comorbidity were each independently associated with a significantly decreased likelihood of being offered chemotherapy.

**Table 4:** Proportion and odds ratios of patients with stage III colon cancer *not *offered chemotherapy

		total	(n, %) not offered chemo	Crude odds ratio	95% confidence intervals	Adjusted odds ratio*	95% confidence intervals
Sex	Female	103	(29, 28%)	1.0		1.0	
	Male	86	(31, 36%)	1.4	0.8–2.7	1.8	0.9–3.9

Age	25–54 yrs	33	(4, 12%)	1.0		1.0	
	55–64 yrs	50	(10, 20%)	1.8	0.5–6.4	1.4	0.4–5.1
	65–74 yrs	55	(14, 26%)	2.5	0.7–8.3	1.2	0.3–4.5
	75+ yrs	51	(32, 63%)	12.2	3.7–40.1	8.7	2.3–32.4

Ethnicity	Non-Maori	105	(31, 30%)	1.0		1.0	
	Maori	84	(29, 35%)	1.3	0.7–2.3	1.5	0.7–3.4

Charlson comorbidity index	O	97	(15, 16%)	1.0		1.0	
	1–2	76	(32, 42%)	4.0	2.0–8.1	3.2	1.4–7.1
	3+	16	(13, 81%)	23.7	6.0–93.3	20.1	4.2–95.6

*adjusted for all listed variables

Not being offered adjuvant chemotherapy was associated with significantly poorer survival among those with stage III disease after adjusting for age, sex and comorbidity (HR = 2.32; 95%CI = 1.34–4.00 for cancer specific survival; and HR = 2.71; 95% CI = 1.68–4.36 for all-cause survival). Furthermore, the survival differential for those with the highest level of comorbidity (Charlson Index score ≥3) compared to those with the lowest (Charlson Index score = 0) was considerably reduced after adjusting for whether or not they were offered adjuvant chemotherapy. After adjustment for age and sex, those with a Charlson score of 3+ compared with 0 had a cancer specific HR of 2.27 (95% CI 0.96–5.45). After adjusting for being offered chemotherapy this HR was 1.43 (95% CI 0.57–3.60), a 66% reduction in excess risk of death. The pattern was similar for all-cause survival with a 59% reduction in excess risk of death (HR = 3.56, 95% CI 1.80–7.05 to HR = 2.06, 95% CI 0.99–4.30).

## Discussion

We found that comorbidity was very common among this nationally representative cohort of patients with colon cancer. Seven out of ten patients had at least one recorded comorbid condition, and comorbidity had a substantial negative impact on both cancer specific and all-cause survival. This impact varied both between different comorbid conditions, and between the two measures of survival used. We found that multivariable models of survival that included specific conditions known to increase mortality hazard by 20% or more had better fit than models using comorbidity count, Charlson index or all comorbid conditions. This effect was particularly pronounced for cancer specific survival. We found that patients with stage III colon cancer who had higher levels of comorbidity or who were older were considerably less likely to be offered adjuvant chemotherapy, despite our finding that being offered chemotherapy improved cancer specific and all-cause survival in these patients.

Our findings are consistent with other work in this area showing that comorbidity is common among people with colon cancer and that it has a substantial impact on survival [2-4,12,15,41-45]. A number of studies have found an association between the Charlson index and survival among colon cancer patients [4,12,14,44,46]. The Charlson index is a well established tool for summarising comorbidity; however there are problems with its use. It includes some conditions that have not been shown to have an impact on survival among patients with colon cancer (e.g. peptic ulcer disease), it may exclude some that do have such an impact (e.g. non-cerebrovascular neurological conditions), and it assumes that the impact of multiple conditions is additive on a relative scale. In fact, Gross et al (2006)[1] found the effects of combinations of comorbidities on survival among colon cancer patients were complex and difficult to predict. For example, diabetes, CHF and chronic respiratory disease all exerted strong independent effects on survival. Patients with both CHF and diabetes had considerably worse survival than those with either condition individually; whereas those with chronic respiratory disease had similar survival rates whether they also had diabetes or not. Consistent with this, we found that inclusion of specific conditions within multivariable models provided better model fit than either a simple comorbidity count or Charlson comorbidity index, and were therefore more likely to adequately adjust for the effect of comorbidity on cancer survival. Baldwin et al (2006) compared four administrative claims-based measures of comorbidity and concluded that while none was clearly superior, the Elixhauser measure, which is based on individual conditions, was possibly best at predicting chemotherapy receipt and non-cancer death[15] Assessing the impact of individual conditions on survival may also be more useful to clinicians in terms of assessing the prognosis of particular patients. However, there are clearly situations where a single summarised measure of comorbidity is required, either because study numbers are not sufficiently large to include individual conditions in analyses, or because the impact of a global measure of comorbidity is of interest, for example, when trying to assess the independent effect of comorbidities adjusting for other covariates.

It is not entirely clear the extent to which comorbidity acts on survival directly or through its impact on treatment choice or effectiveness. Intuitively it is likely that both play a part. We found clinicians were less likely to offer chemotherapy to patients with higher levels of comorbidity and older patients consistent with evidence from other studies [11,15,25,27,32,46]. There are a number of possible explanations for this pattern including physicians' concern regarding the potential for increased toxicity of treatment among these groups, concern that these patients may have a short remaining life expectancy limiting the benefit of adjuvant therapy or concern that these treatments may be less effective in elderly or sicker patients. In fact, there is good evidence that these treatments are both well tolerated and effective even in very elderly patients, and that elderly patients are as likely to choose chemotherapy as younger ones [26-28,33,35]. There is less evidence relating to these factors in patients with multiple comorbidity. However, the few available studies suggest that chemotherapy has a positive effect on survival among patients with stage III colon cancer even among those with high comorbidity [1,46]. We also found being offered chemotherapy was associated with a reduction in excess mortality risk of around 60% for both cancer specific and all-cause mortality among those with highest levels of comorbidity. This again supports the idea that at least part of the survival disparity between those with and without comorbidity is mediated through differences in treatments offered [7]. It is important to note that our analyses are based on observational data, not randomised trial. Therefore, it is likely that the variable 'being offered chemotherapy' may be acting as a surrogate for other prognostic factors on which we did not have data such as functional status. This means that the reduction in mortality associated with the offer of chemotherapy may be somewhat lower in reality than the 60% observed in this study.

Potential limitations of our study include limited power and retrospective ascertainment of comorbidity. Our sample was too small to assess the impact of rarer conditions on survival, or to assess the impact of multiple combinations of specific conditions. Also, given that this was retrospective, observational data, conditions were only included if they had been diagnosed by a physician at the time of diagnosis. This may have led to some misclassification in those with undiagnosed disease, which would most likely dilute the apparent effect of comorbidity on survival.

A principal strength of our study is the presence of detailed comorbidity data based on clinical records rather than administrative data. Comorbidity data were extracted from clinical notes by a study physician, allowing comprehensive assessment and classification of patient comorbidity without restriction to pre-defined categories. Comorbidity data from medical notes have several advantages over data obtained from administrative sources in which there tends to be under ascertainment of some conditions and there can be difficulty differentiating complications of treatment from pre-existing conditions [2,16,47]. Also, because we had access to clinical notes, we were able to ascertain the proportion of Stage III patients who were offered chemotherapy, rather than those who received it. The offer of chemotherapy is important in identifying the clinical decisions and preferences of physicians. Full data were obtained for a high proportion of our cohort (86.0% of eligible cases).

## Conclusion

Comorbidity is an important factor in the assessment of mortality risk for patients with colon cancer. The measurement and application of comorbidity in studies of cancer survival remains problematic. Based on our findings we recommend using data on individual conditions where possible in models that aim to adjust for comorbidity as a confounder or to assess its role as a mediating influence in survival. However in studies where comorbidity is a primary exposure or outcome of interest it may still be necessary to use global measures of comorbidity such as comorbidity count or a weighted index. Our findings also support the idea that the poorer prognosis of those with high comorbidity levels is at least partially mediated by less active treatment in these patients. We found clear evidence that chemotherapy has a positive impact on survival among stage III patients, even in those with the highest levels of comorbidity. Cancer treatment guidelines should ensure patients are offered all appropriate treatment and that chemotherapy is not withheld unnecessarily in older patients or those with higher comorbidity. Further research would be useful to identify which patients with comorbidity are most likely to benefit from additional treatment, and what criteria should be used to decide whether adjuvant treatment should be appropriately withheld.

## Competing interests

The authors declare that they have no competing interests.

## Authors' contributions

DS initiated and led the study design, the data analysis and interpretation, and wrote the first draft of the paper. SH contributed to study design, collected the data, contributed to the data analysis and interpretation, and draft revisions. TB contributed to study conception and design, data analysis and interpretation, and draft revisions. BR contributed to study design, data interpretation, and draft revisions. GP contributed to study design, data analysis and interpretation, and draft revisions. LD provided clinical advice, contributed to study design, data interpretation, and draft revisions. DC contributed to study design, data interpretation, and draft revisions. KD contributed to study design, data interpretation, and draft revisions. All authors read and approved the final manuscript.

## Pre-publication history

The pre-publication history for this paper can be accessed here:

http://www.biomedcentral.com/1471-2407/9/116/prepub
